# Pterostilbene alleviates pulmonary fibrosis by regulating ASIC2

**DOI:** 10.1186/s13020-021-00474-7

**Published:** 2021-07-28

**Authors:** Yanfang Peng, Yingwen Zhang, Yabing Zhang, Xiuping Wang, Yukun Xia

**Affiliations:** Department of Traditional Chinese Medicine, Zhongnan Hospital of Wuhan University, No. 169 Donghu Road, Wuchang District, Wuhan, 430071 Hubei China

**Keywords:** Idiopathic pulmonary fibrosis, TGF-β1, Epithelial-mesenchymal transition, Death, Pterostilbene, ASIC2

## Abstract

**Background:**

Idiopathic pulmonary fibrosis (IPF) is a serious chronic disease of the respiratory system, but its current treatment has certain shortcomings and adverse effects. In this study, we evaluate the antifibrotic activity of pterostilbene (PTE) using an in vitro IPF model induced by transforming growth factor (TGF)-β1.

**Methods:**

A549 and alveolar epithelial cells (AECs) were incubated with 10 ng/ml TGF-β1 to induce lung fibroblast activation. Then, 30 μmol/L of PTE was used to treat these cells. The epithelial–mesenchymal transition (EMT), extracellular matrix (ECM) accumulation, and autophagy in cells were evaluated by western blot. Apoptosis was validated by flow cytometry analysis and western blot. Transcriptome high-throughput sequencing was performed on A549 cells incubated with TGF-β1 alone or TGF-β1 and PTE (TGF-β1 + PTE), and differentially expressed genes in PTE-treated cells were identified. The acid sensing ion channel subunit 2 (ASIC2) overexpression plasmid was used to rescue the protein levels of ASIC2 in A549 and AECs.

**Results:**

TGF-β1 caused EMT and ECM accumulation, and blocked the autophagy and apoptosis of A549 and AECs. Most importantly, 30 μmol/L of PTE inhibited pulmonary fibrosis induced by TGF-β1. Compared with TGF-β1, PTE inhibited EMT and ECM accumulation and rescued cell apoptosis and autophagy. The results of transcriptome high-throughput sequencing revealed that PTE greatly reduced the protein level of ASIC2. Compared with the TGF-β1 + PTE group, the transfection of ASIC2 overexpression plasmid stimulated the EMT and ECM accumulation and inhibited apoptosis and autophagy, suggesting that PTE inhibited pulmonary fibrosis by downregulating ASIC2.

**Conclusions:**

This study suggests that PTE and ASIC2 inhibitors may have potential as IPF treatments in the future.

## Background

Idiopathic pulmonary fibrosis (IPF) is a chronic, progressive, and fibrotic lung disease characterized by sustained reproduction of lung fibroblasts and accumulation of extracellular matrix (ECM), ultimately leading to respiratory system failure and death [1, 2]. IPF occurs worldwide, with an increasing prevalence [3]. Although, it is unclear whether such an increase is caused by improved recognition of the disease or by an actual increase in morbidity. IPF is more common in men and people with a history of smoking [4]. As a typical senile disease, the median age at diagnosis is 65 years [5, 6]. The disease course is uncertain and unpredictable, and prognosis is extremely poor. The median survival of patients after diagnosis is 3–5 years. Currently, lung transplantation remains as the only treatment that can significantly improve the survival rate of carefully selected patients [7]. Although, two molecules have been approved by the FDA to treat IPF, namely, pirfenidone and nintedanib [8, 9]. These drugs have been shown to slow down the declining lung function in patients with IPF, but they are ineffective in reversing the process of fibrosis or even stabilizing lung function and cannot improve the survival rate or the quality of life of patients [10, 11].

The main feature of IPF is the accumulation of fibroblasts. Fibroblasts respond to secreted transforming growth factor-β1 (TGF-β1) and differentiate into myofibroblasts [12]. Myofibroblasts express α-smooth muscle actin (α-SMA), and large amounts of ECM (such as collagen) accumulate in the fibrous hyperplasia foci to inhibit cell exchange. In IPF, the scattered accumulation of myofibroblasts in fibrotic lesions and the deposition of ECM lead to irreversible destruction of lung structure, respiratory failure, and death [12]. TGF-β1 treatment of human type II alveolar epithelial cells (AECs) in vitro can induce fibrosis.

**Fig. 1:**
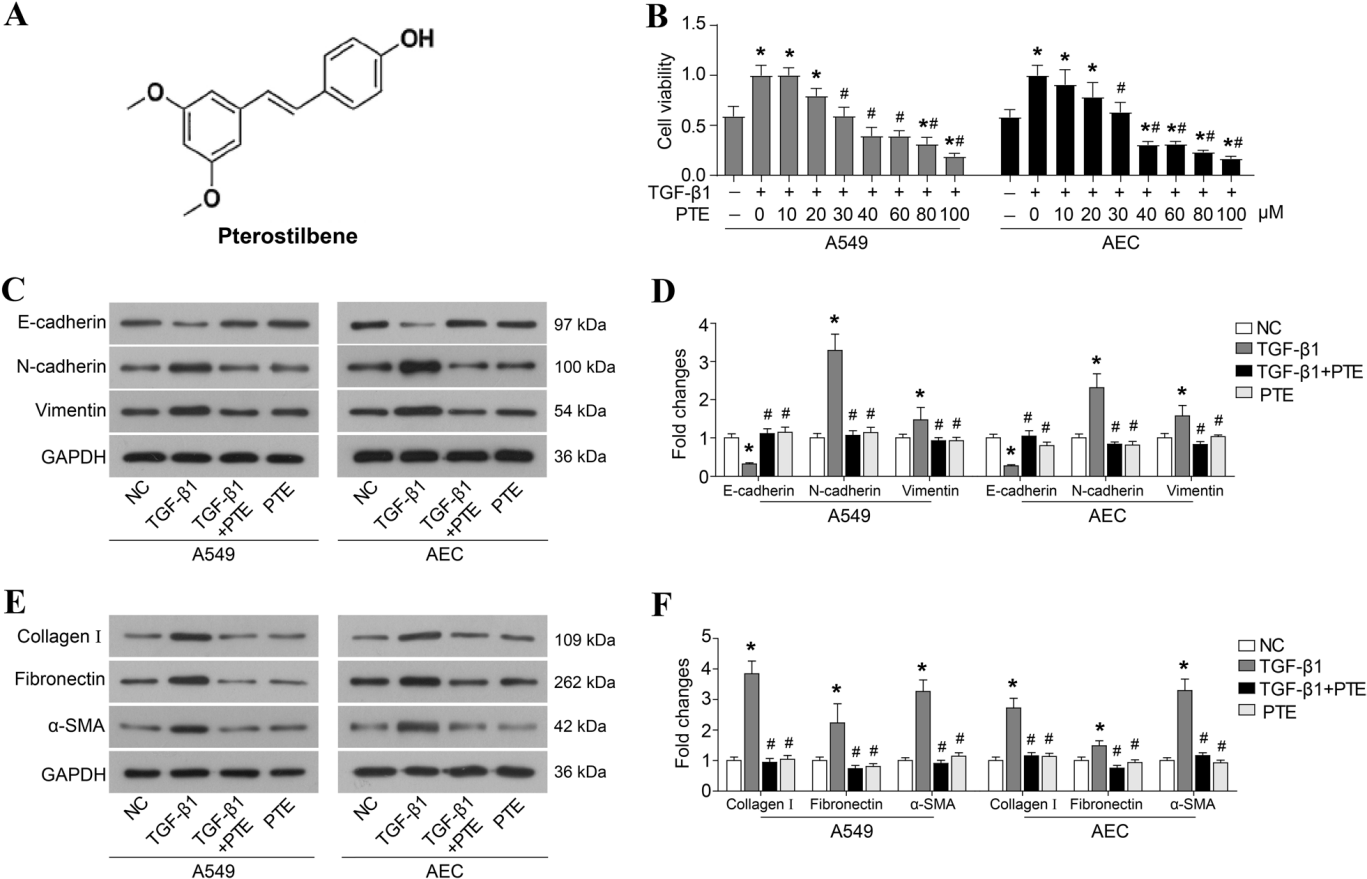
PTE inhibits TGF-β1-induced cell proliferation, EMT, and ECM accumulation. **A** Chemical structure of PTE. **B** A549 and AECs were treated with 10 ng/ml TGF-β1 and various concentrations of PTE (0, 10, 20, 30, 40, 60, 80, and 100 μmol/L) for 24 h, and cell viability was detected by CCK8 assay. A549 and AECs were treated with 10 ng/ml TGF-β1 and 30 μmol/L PTE for 24 h. The key protein of EMT (**C**, **D**), α-SMA, and ECM (**E**, **F**) were detected by western blot. All experiments were repeated three times independently, and Student’s t test or ANOVA was used to compare the differences between groups. *P < 0.05 compared with the NC group; ^#^P < 0.05 compared with the TGF-β1 group

Numerous studies have reported that naturally active compounds isolated from plants or herbs have the potential to treat organ fibrosis, including pulmonary fibrosis [13–15]. Pterostilbene (PTE; trans-3,5-dimethoxy-4-hydroxystilbene) (Fig. 1A) is a structural analog of resveratrol, which is mainly derived from the genus *Pterocarpus* of plants, such as grapes and blueberry [16]. PTE was reported to possess anti-oxidation, anti-inflammatory, anticancer, antihypertensive, and anti-aging properties [17, 18]. Emerging evidence also shows that PTE can intervene in the fibrosis process of various organs. PTE can attenuate liver and renal fibrosis induced by excessive fructose intake by inhibiting TGF-β1/Smads signal transduction [19, 20]. It can also reduce fructose-induced myocardial fibrosis by inhibiting the Pitx2c/miR-15b pathway driven by reactive oxygen species [21]. Moreover, PTE alleviates renal fibrosis in a mouse model of severe hyperuricemia nephropathy by inhibiting the activation of TGF-β1/Smad3, Src and STAT3 signaling pathways [22, 23]. In a previous study, we injected bleomycin intratracheally to induce pulmonary fibrosis in SD rats and found that PTE (30 mg/(kg/d)) can reduce the severity of bleomycin-induced pulmonary fibrosis [24, 25].

Thus, in the present study, we aimed to evaluate the antifibrotic effect of PTE on the IPF process induced by TGF-β1 and investigate the underlying mechanism.

## Materials and methods

### Cell culture and treatment

Human normal alveolar epithelial cells AECs and epithelioid lung cancer cells A549 were purchased from the American Type Culture Collection (ATCC; Manassas, VA) and cultured in Dulbecco’s Modified Eagle Medium (HyClone, Logan, UT) containing 10% fetal bovine serum (Gibco, Thermo Fisher Scientific, Inc., Waltham, MA) in a humidified atmosphere of 5% CO_2_ at 37 °C. The cells were cultured in serum-free medium for 12 h for synchronization and further incubated with TGF-β1 (10 ng/ml; R&D Systems, Minneapolis, MN) to induce lung fibroblast activation. PTE (Sigma-Aldrich, St. Louis, MO) was dissolved in DMSO and further diluted before use. The cells were treated with TGF-β1, TGF-β1 with PTE (TGF-β1 + PTE), and PTE for 24 h, and untreated cells were used as negative control (NC). pcDNA3.1-ASIC2 plasmid was constructed and transfected into cells using Lipofectamine 2000 (Invitrogen, Thermo Fisher Scientific, Inc.) to induce overexpression of acid sensing ion channel subunit 2 (ASIC2).

### Dose-dependent assay

Cells were planted in 96-well plates at a density of 5 × 10^3^ cells per well and treated with 10 ng/ml TGF-β1 and various concentrations of PTE (0, 10, 20, 30, 40, 60, 80, and 100 μmol/L) for 24 h. Then, cells were incubated with CCK8 reagent (10 μl/well; Beijing Solarbio Science & Technology Co., Ltd., Beijing, China) for another 1.5 h. Finally, the optical density (OD) value at 450 nm of each well was detected using a microplate reader.

### Western blot analysis

Cells were collected and lysed in RIPA (Beyotime Biotechnology, Shanghai, China), and the extracted protein concentration was determined using BCA kit (Beyotiome). Proteins of each sample were separated with 10% SDS-PAGE and then transferred onto PVDF membranes (Bio-Rad Laboratories, Hercules, CA). After blocking in 5% milk for 1 h at room temperature, the membranes were incubated with primary antibodies at 4 °C overnight and secondary antibodies for 2 h at room temperature. The blots were visualized using an enhanced chemiluminescence kit (CWBIO, Beijing, China) and quantified by ImageJ software (NIH, Bethesda, MD).

The primary antibodies used in this study were as follows: E-cadherin (3195, Cell Signaling Technology, Inc., Danvers, MA), N-cadherin (207608, Abcam, Cambridge, UK), vimentin (5741, Cell Signaling Technology, Inc.), Collagen I (sc-293, Santa Cruz Biotechnology, Dallas, TX), fibronectin (15613–1-AP, ProteinTech Group, Rosemont, IL), α-SMA (5694, Abcam), Bcl2 (ab32124, Abcam), Bax (ab32503, Abcam), cleaved caspase-3 (ab13847, Abcam), LC3-I/II (14600-1-AP, ProteinTech Group), Beclin1 (ab207612, Abcam), p62 (18420-1-AP, ProteinTech Group), p21 (ab109520, Abcam), ASIC2 (ab169768, Abcam), and GAPDH (60004-1-Ig, ProteinTech Group).

### Flow cytometry assay

Cell apoptosis was measured using the Annexin-V-FITC/PI kit (BioVision, CA) according to the instructions. Cells (1 × 10^5^) treated with TGF-β1 or PTE for 24 h were cultured in serum-free medium for another 24 h and stained with Annexin-V-FITC/PI for 10 min in the dark. Subsequently, the rate of apoptotic cells was detected using a flow cytometer (BD FACSCanto II, BD Biosciences, Franklin Lakes, NJ) and analyzed using the FlowJo software (Tree Star, USA).

### High-throughput sequencing

Transcriptome high-throughput sequencing was performed on A549 cells incubated with TGF-β1 (TGF-β1) alone or TGF-β1 and PTE (TGF-β1 + PTE) for 24 h. Cross-linked RNA fragments were isolated and converted into cDNA libraries, and high-throughput sequencing was performed with Illumina Hiseq (San Diego, CA). The original image data file obtained by sequencing was converted into raw data by CASAVA base calling analysis. Raw data were filtered to get clean reads. The clean reads were aligned to a reference genome using HISAT [26]. The PossionDis algorithm was used for differential gene detection. |log2(Fold Change)|> 1 & q value < 0.001 genes are regarded as differentially expressed genes.

### Statistical analysis

Data were expressed as mean ± SD from three separate experiments and analyzed using GraphPad Prism 7.0 (GraphPad Software Inc., La Jolla, CA). Student’s t test or one-way analysis of variance (ANOVA) was used to compare the differences between groups. P < 0.05 was considered significant.

## Results

### PTE inhibits TGF-β1-induced cell proliferation, EMT, and ECM accumulation

The chemical structure of PTE is shown in Fig. 1A. As exhibited in Fig. 1B, PTE at a dose of 30–100 μmol/L significantly reduced the cell viability of 10 ng/ml TGF-β1-exposed A549 and AECs (Fig. 1B). Furthermore, the preliminary experiments showed that 10 ng/ml TGF-β1 caused EMT after 48 h of exposure of A549 and AECs (Fig. 1C, D). The expressions of N-cadherin and Vimentin in cells significantly increased after incubation with 10 ng/ml TGF-β1 (Fig. 1C, D), while expressions of E-cadherin protein decreased (Fig. 1C and D). Moreover, 30 μmol/L of PTE suppressed expressions of N-cadherin and Vimentin and rescued E-cadherin expression in TGF-β1-exposed cells (Fig. 1C, D). As regards ECM accumulation, the results from the western blot analysis confirmed the increased protein levels of α-SMA and fibronectin, as well as collagen 1, which was induced by TGF-β1 in A549 and AECs (Fig. 1E, F). PTE significantly reduced the expressions of α-SMA, fibronectin, and collagen 1 (Fig. 1E, F). In addition, incubation with PTE alone, without TGIF1, appeared to have no effect on EMT and ECM accumulation when compared with normally cultured A549 and AECs (Fig. 1C–F).

### PTE promotes apoptosis and autophagy in TGF-β1-induced cells

**Fig. 2:**
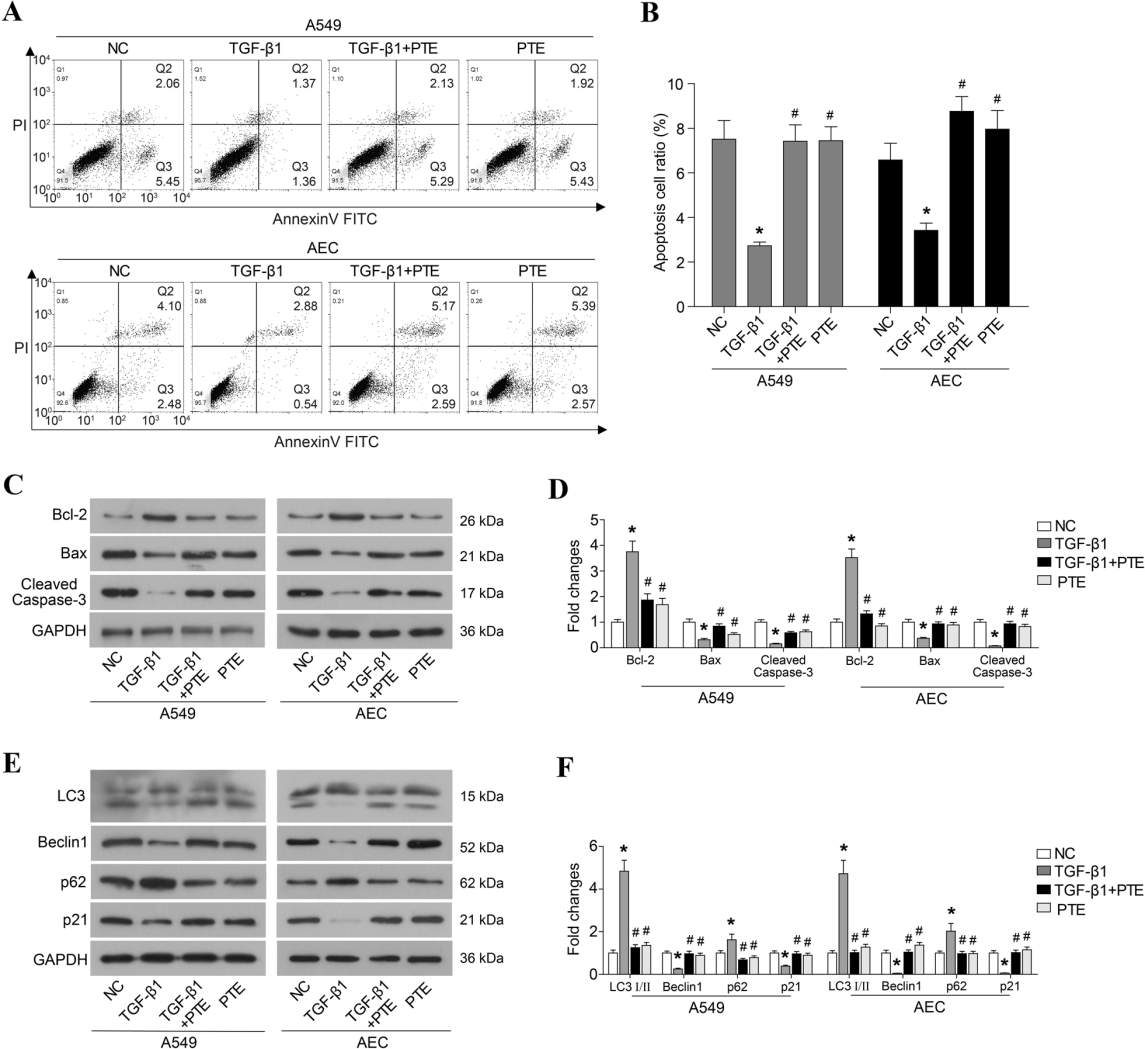
PTE promotes apoptosis and autophagy in TGF-β1-induced cells. A549 and AECs were treated with 10 ng/ml TGF-β1 and 30 μmol/L PTE for 24 h, and apoptosis was analyzed by flow cytometry assay (**A,** **B**). The expression of apoptosis-related protein (**C**, **D**) and autophagy-related protein (**E**, **F**) were detected by western blot. All experiments were repeated three times independently, and Student’s t test or ANOVA was used to compare the differences between groups. *P < 0.05 compared with the NC group; ^#^P < 0.05 compared with the TGF-β1 group

Some studies have reported that PTE induces apoptosis in ovarian [27] and pancreatic cancer cells [28]. In this study, treatment with TGF-β1 significantly decreased the proportion of apoptotic cells (Fig. 2A, B), inhibited the expressions of Bax and cleaved caspase-3, and induced the expression of Bcl2 (Fig. 2C, D). The treatment with PTE rescued the apoptosis that was inhibited by TGF-β1 (Fig. 2A, B), elevated Bax and cleaved caspase-3 protein levels, and degraded Bcl2 in A549 and AECs (Fig. 2C, D). In addition, the effect of PTE in cell autophagy was evaluated. As shown in Fig. 2E and F, TGF-β1 significantly inhibited expressions of LC3-II, Beclin-1 and p21 proteins and enhanced expressions of LC3-I and Beclin-1 protein in A549 and AECs, which all were reversed by PTE. Moreover, incubation with PTE alone, without TGIF1, had no effect on the apoptosis and autophagy when compared with normally cultured A549 and AECs (Fig. 2).

### Identification of differentially expressed genes in PTE-treated cells

**Fig. 3:**
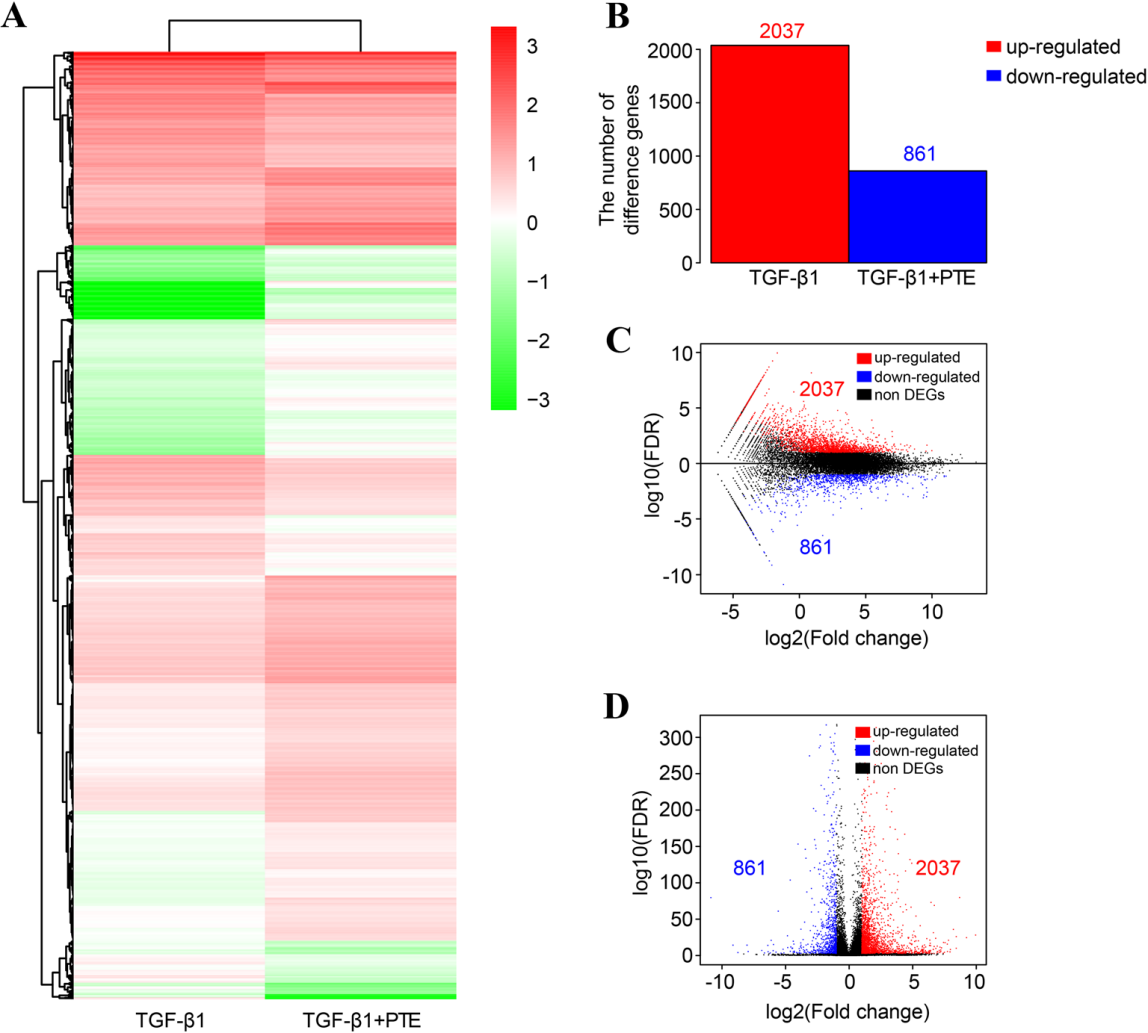
Identification of differentially expressed genes in PTE-treated cells. Transcriptome high-throughput sequencing was performed on A549 cells incubated with TGF-β1 alone (TGF-β1) or with TGF-β1 and PTE (TGF-β1 + PTE) for 24 h. **A** Clustering heat map of differentially expressed genes. **B** Statistical graph of the number of differential genes. **C** Volcano map of differentially expressed genes. **D** MA map of differentially expressed genes

Transcriptome high-throughput sequencing was also performed on A549 cells incubated with TGF-β1 alone (TGF-β1) or with TGF-β1 and PTE (TGF-β1 + PTE) for 24 h to identify differentially expressed genes caused by PTE. TGF-β1 samples produced 8.4430 Gb clean bases, 95.74% reads were compared with the reference genome, and 14848 genes were measured. TGF-β1 + PTE samples produced 8.4045 Gb clean bases, 95.54% reads were compared with the reference genome, and 15451 genes were measured. Compared with PTE samples, 2898 differentially expressed genes were detected in TGF-β1 + PTE samples. Among them, 2037 genes were differentially upregulated and 861 genes were differentially downregulated (Fig. 3).

### PTE inhibits pulmonary fibrosis by downregulating ASIC2

**Fig. 4:**
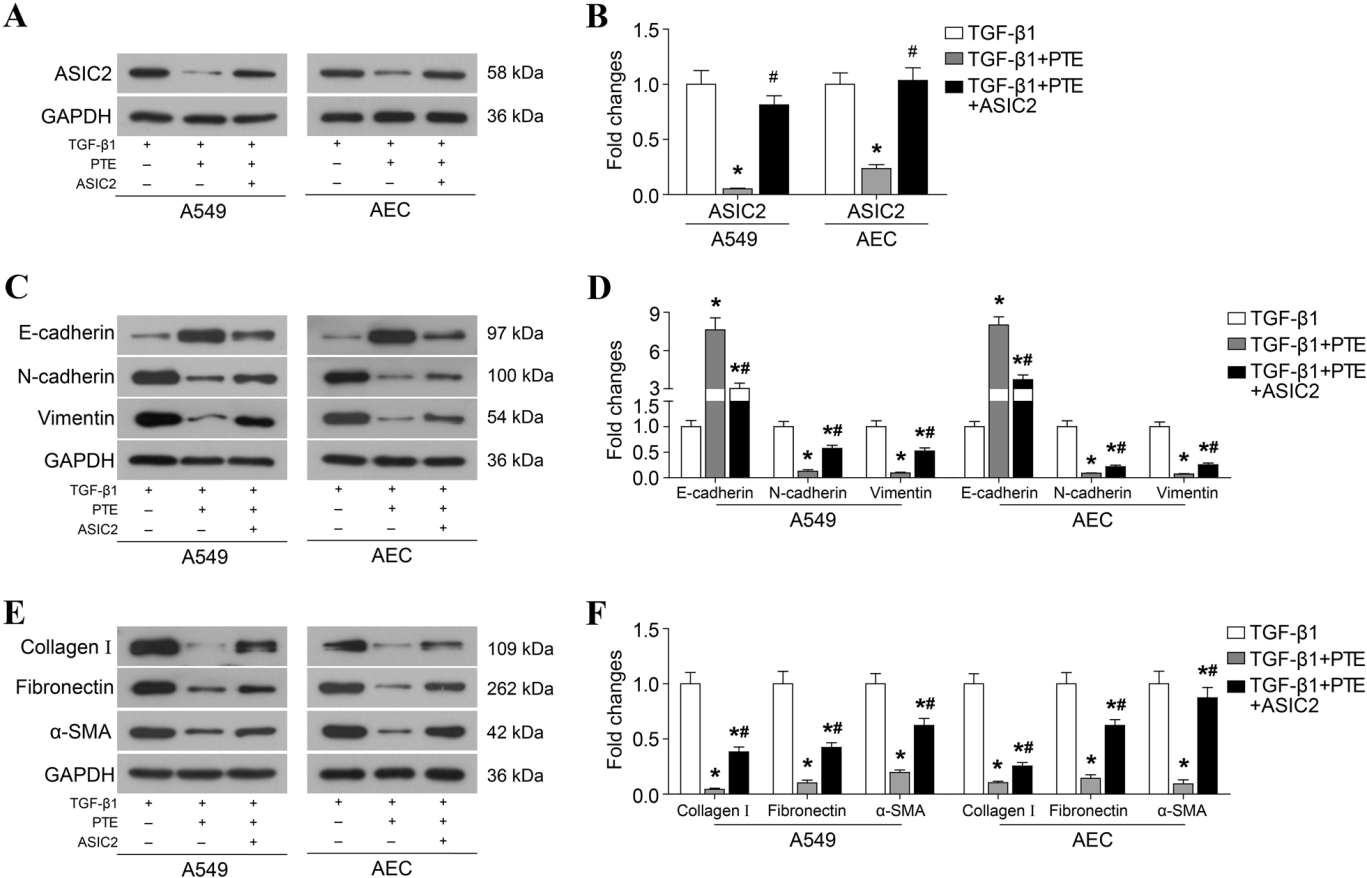
PTE inhibits TGF-β1-induced EMT and ECM accumulation by downregulating ASIC2. A549 and AECs that were transfected with ASIC2 overexpression plasmid were treated with 10 ng/ml TGF-β1 and 30 μmol/L PTE for 24 h. The expressions of ASIC2 (**A**, **B**), key protein of EMT (**C**, **D**), α-SMA, and ECM (**E,** **F**) were detected by western blot. All experiments were repeated 3 times independently, and Student’s t test or ANOVA was used to compare the differences between groups. *P < 0.05 compared with TGF-β1 group; ^#^P < 0.05 compared with TGF-β1 + PTE group

**Fig. 5:**
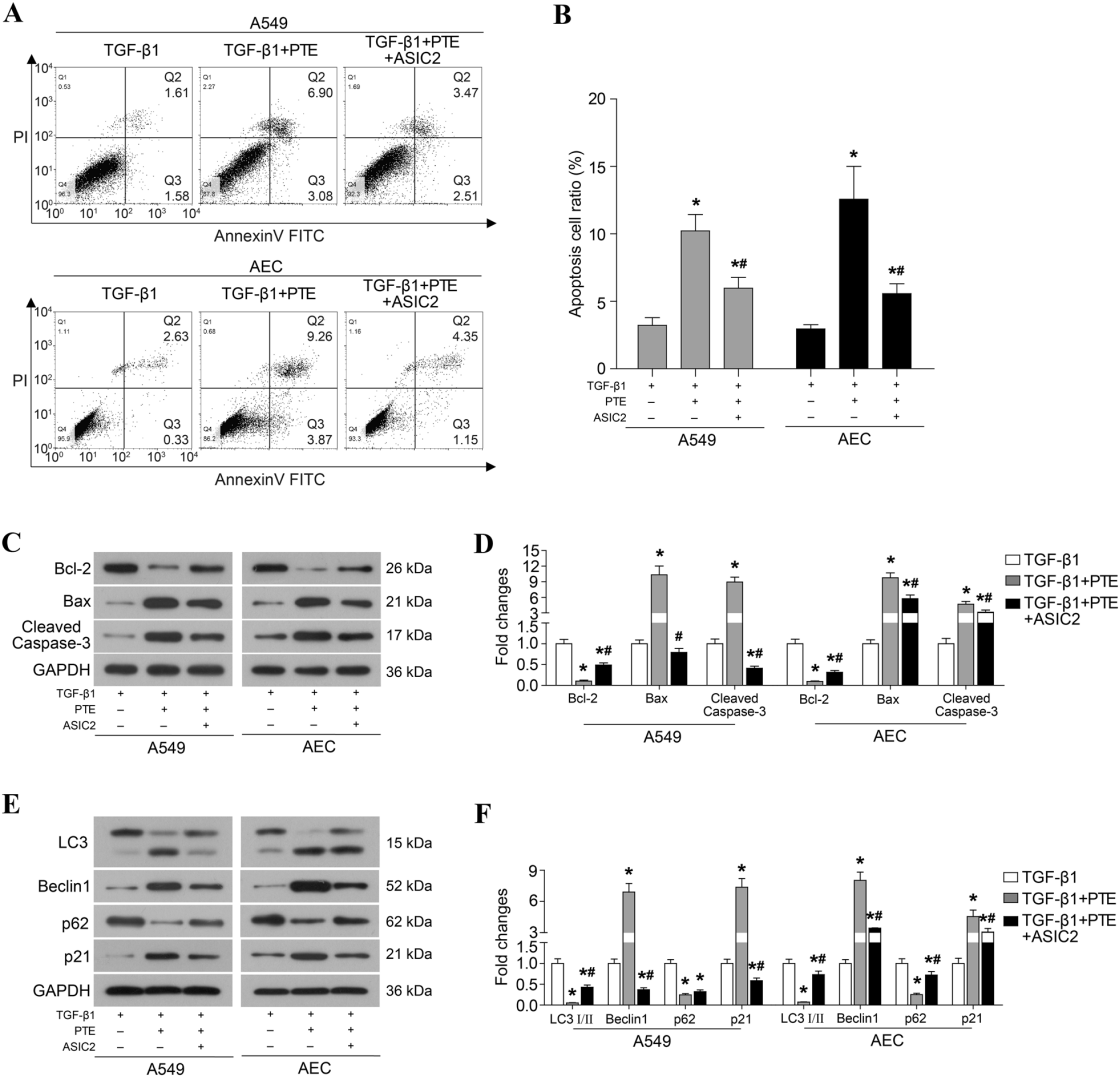
PTE promotes apoptosis and autophagy by downregulating ASIC2 in TGF-β1-induced cells. A549 and AECs that were transfected with ASIC2 overexpression plasmid were treated with 10 ng/ml TGF-β1 and 30 μmol/L PTE for 24 h. Apoptosis was analyzed by flow cytometry assay (**A**, **B**). The expressions of apoptosis-related protein (**C**, **D**) and autophagy-related protein (**E**, **F**) were detected by western blot. All experiments were repeated 3 times independently, and Student’s t test or ANOVA was used to compare the differences between groups. *P < 0.05 compared with TGF-β1 group; ^#^P < 0.05 compared with TGF-β1 + PTE group

Among the 861 differentially downregulated genes, PTE inhibited pulmonary fibrosis by downregulating ASIC2. Compared with TGF-β1, PTE significantly reduced the protein levels of ASIC2 (Fig. 4A, B). To explore the role of ASIC2, the ASIC2 overexpression plasmid was used to rescue the protein levels of ASIC2. Furthermore, the restoration of ASIC2 protein levels stimulated EMT (Fig. 4C and D) and ECM accumulation (Fig. 4E, F) and inhibited apoptosis and autophagy (Fig. 5) compared with the TGF-β1 + PTE group.

## Discussion

The results of this study showed that PTE suppressed TGF-β1-induced cell proliferation, EMT, and ECM accumulation and promoted TGF-β1-inhibited autophagy and apoptosis. Moreover, our data showed that ASIC2 as a downstream effector of PTE is involved in the antifibrotic action of PTE on IPF.

TGF-β1 is considered the master profibrotic cytokine in the process of fibrosis, which is commonly used to induce cellular pulmonary fibrosis to establish an in vitro IPF model [29, 30]. TGF-β1 can stimulate the abnormal proliferation of lung fibroblasts, promote the formation of myofibroblasts and activation of pro-fibrotic gene, leading to excessive accumulation of ECM between the lung interstitium and alveoli [31]. TGF-β1 is also the principal driver of fibrogenesis, a dynamic pathophysiologic process that involves cell injury and apoptosis [32]. In this study, western blot analysis revealed that exposing A549 and AECs to 10 ng/ml TGF-β1 caused EMT and ECM accumulation in cells. TGF-β1 also impeded the autophagy and apoptosis of A549 and AECs, which was proved by the results of both flow cytometry analysis and western blot analysis. Most importantly, we found that PTE inhibited pulmonary fibrosis induced by TGF-β1. Compared with TGF-β1, PTE inhibited EMT and ECM accumulation and rescued the apoptosis and autophagy in cells.

We further performed transcriptome high-throughput sequencing on A549 cells incubated with TGF-β1 alone or TGF-β1 and PTE and identified differentially expressed genes caused by PTE. We found that PTE significantly reduced the protein levels of ASIC2. Furthermore, the ASIC2 overexpression plasmid was used to rescue the protein levels of ASIC2 in A549 and AECs, which stimulated EMT and ECM accumulation and inhibited apoptosis and autophagy when compared with the TGF-β1 + PTE group, suggesting that PTE inhibited pulmonary fibrosis by downregulating ASIC2.

ASICs are a group of proton-gated ion channels belonging to the degenerin/epithelial sodium channel (DED/ENaC) family, which can be activated by extracellular protons. Although the mechanisms of cellular metabolism during IPF are still poorly understood, lactate has recently been identified as a metabolite that is elevated in the lung tissue of patients with IPF [33]. Moreover, acidification or low pH can activate ASICs [34]. ASICs are encoded by four genes (*ASIC1-4*). These genes generate 6 subtypes, including ASIC1a, ASIC1b, ASIC2a, ASIC2b, ASIC3, and ASIC4, through alternative splicing. Each subtype has different biophysical properties [35]. A preliminary study suggested that ASICs are widely distributed in the central and peripheral nervous system [36]. However, a study showed that multiple subunits of ASICs are also present in the vascular smooth muscles and endothelial cells of various vascular beds [37]. Su et al. reported on the expressions of ASIC2 and ASIC3 in human submucosal (Calu-3), bronchial (16HBE14o), pancreas (CFPAC), and colon (T84) epithelial cells as well as the expression of lung tissue slices in alveolar tissue [38]. A study also determined the expressions of ASIC1, ASIC2, and ASIC3 in A549 cells [39]. An acidic extracellular medium or overexpression of ASIC1a promotes the proliferation and migration of A549 cells [39].

Ion channels are transmembrane proteins that have physiological and pathological functions across biological membranes. Currently, approximately 13% of drugs used to treat various human diseases, including cardiovascular and neurological diseases, are mainly aimed at ion channels [40]. These channels are associated with many human diseases/pathologies, including IPF. For example, the classical transient receptor potential 6 is a cation channel permeable to Na^+^ and Ca^2+^, which can promote the differentiation of primary mouse lung fibroblasts into myofibroblasts. Its specific channel inhibitors may help in improving future treatment of IPF [41]. Thus, our results suggest that ASIC2 inhibitors may have potential in the development of IPF treatments in the future.

## Conclusion

In summary, data obtained using an in vitro IPF model demonstrated that PTE could inhibit TGF-β1-induced EMT and ECM accumulation and promote autophagy and apoptosis, suggesting that PTE may be served as a promising strategy for IPF treatment. Moreover, we found that ASIC2 as a downstream effector of PTE may be involved in the activity of PTE to alleviate pulmonary fibrosis. Further investigation of the activity and mechanism of PTE in vivo are still required.

## Data Availability

The datasets generated and/or analysed during the current study are not publicly available, but are available from the corresponding author on reasonable request.
